# Prospective associations of physical fitness with spinal flexibility in childhood: implications for primary prevention of non-specific back pain

**DOI:** 10.3389/fped.2023.1180690

**Published:** 2023-07-04

**Authors:** Svea Bade, Giulia Lona, Denis Infanger, Katharina Endes, Ralf Roth, Oliver Faude, Henner Hanssen

**Affiliations:** Department of Sport, Exercise and Health, Medical Faculty, University of Basel, Basel, Switzerland

**Keywords:** childhood, back pain, spinal flexibility, physical fitness, prevention

## Abstract

**Objectives:**

Early predictors for back pain need to be identified for the development of prevention strategies starting as early as childhood. For this purpose, the relationship between physical fitness and spinal flexibility at the age of six years and its prediction for the development of non-specific back pain (BP) during childhood were analyzed.

**Methods:**

In this prospective cohort study with 4-year follow-up, school children from the Swiss Canton Basel-Stadt, aged 6–8 (2014) at baseline and 10–12 years (2018) at follow-up, were recruited from 26 primary schools (*n* = 238) within a mandatory evaluation of motor skills. Data for spinal flexibility were collected by use of a hand-held computer-assisted device and physical fitness was assessed by shuttle run performance at both time points. Occurrence of non-specific BP was determined by use of a questionnaire at follow-up.

**Results:**

Children with higher physical fitness at baseline achieved a better spinal flexibility four years later (*β* [95% CI] 3.75 [2.19–5.3] degree per 1 stage increase, *p* < 0.001). Higher spinal flexibility by 1 degree at baseline was associated with 2% less odds for non-specific BP at follow-up (OR [95% CI] 0.98 [0.97–0.99] per 1 degree increase, *p* = 0.032). There was little evidence for a direct association between physical fitness at baseline and development of non-specific BP at follow-up (OR [95% CI] 1.13 [0.96–1.34] per 1 stage increase, *p* = 0.128).

**Conclusion:**

Fitness performance is associated with the development of better childhood spinal flexibility over four years. Moreover, a better spinal flexibility at baseline was associated with less non-specific BP at follow-up. This study suggests that physical fitness may be a key modulator of spinal flexibility which itself is a main determinant of non-specific BP during childhood development. Further long-term studies are warranted to confirm our assumptions and to prove trajectories into adolescents and adulthood.

## Introduction

Low back pain (BP) is a worldwide health hazard (1) and the world's leading cause of years lived with disability with the highest prevalence in Western Europe (2). The high costs involved are a socioeconomic healthcare burden (3). In Switzerland, low BP has been reported to account for 6.1% of total health costs (4). The occurrence of initial BP can already be observed in childhood and the prevalence is increasing with age (5–7). BP in childhood (8) and adolescence (9, 10) has been shown to predict development of BP in adulthood. Therefore, it is important to define early predictors of BP in childhood (9, 11). In most cases, the cause of BP cannot be verified (12) and thus most treatment strategies are symptom-orientated (13). Non-specific BP predominates in children and adults alike (14). Risk factors for the development of BP in childhood are, among others, female sex (7, 11) and socioeconomic status (15). Overweight and obesity also play a key role in terms of musculoskeletal pain in children (16, 17). Further, psychosocial factors are related to BP (8), but it is not clear whether it is cause or effect. Wedderkopp et al. (18) pointed out, that higher levels of physical activity during childhood may protect against development of BP in adolescence. It remains unclear whether low physical activity and fitness lead to the development of non-specific BP in childhood and adolescence. In a cross-sectional approach, we have previously demonstrated the association of low physical activity and fitness with reduced spinal flexibility in young children (19). A recent systematic review (10) suggested that the most relevant risk factor for BP in emerging adulthood was the history of BP, which underlines the importance of determining predisposing factors to prevent episodes of BP early in life. Prospective cohort studies are needed to investigate the incidence of infantile BP in relation to underlying risk factors (20–22). Therefore, this study aimed to investigate the longitudinal association between physical fitness and spinal flexibility in relation to and as a predictor for the development of non-specific BP in prepubertal children after four years of follow-up.

## Methods

### Design

In this prospective four-year follow-up cohort study, children were recruited from the Sportcheck study within a mandatory evaluation of physical fitness and motor skills. The study was carried out on behalf of the Cantonal Office of Sports of the City of Basel during regular school classes. All parents of the participants gave written informed consent before data collection. Support for the study was provided by the Department of Education of the City of Basel, the Swiss National Science Foundation (32003B_176172/1) and the Voluntary Academic Society Basel. The Ethics Committee of North-West and Central Switzerland (EKNZ) approved this study (EZNZ No.: 258/12).

### Participants

**Figure 1:**
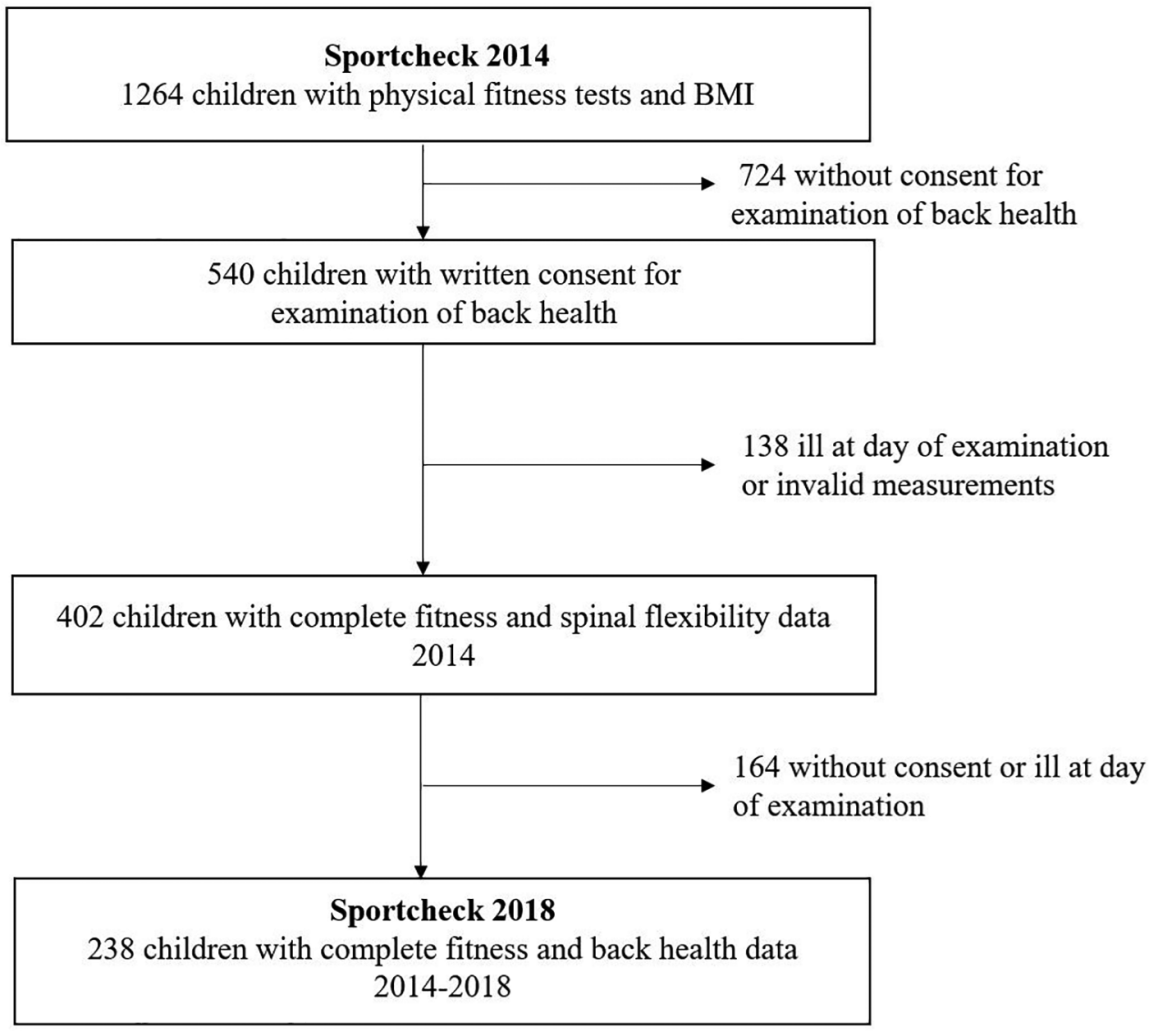
Flow chart of the follow-up cohort.

In 2014, 1,264 first graders were invited to take part in the study and finally, we obtained 402 data sets with complete data for physical fitness, spinal flexibility and BP at baseline. At follow-up in 2018, 238 children were available with complete data at baseline and follow-up (Figure 1).

### Patient and public involvement

The Department of Education of the City of Basel was involved in the conception of the study and represented the interests of the families. Support was given for conducting the study during regular school hours in school settings in order to make the study as barrier-free as possible. Further, the department advocated a feedback loop with the results and individual recommendations for physical activity behavior being sent to all families.

### Measurements

All measurements were conducted on-site at the schools and in the morning. The mandatory fitness test and anthropometric measurements during physical education classes were performed by qualified sports scientists. The examination of the spine was assessed by a single experienced physiotherapist in a separate room.

#### Spinal flexibility

We measured the spinal flexibility by a hand-held, non-invasive and computer-assisted device, the MediMouse (Idiag, Fehraltorf, Switzerland) (23). It is an easily applicable device suitable for children to objectively examine spinal flexibility. The MediMouse has shown a high correlation with the method of x-ray imaging for the assessment of spinal flexibility (*r* = 0.93) (24). The measurements of flexibility in the sagittal plane as applied in this study showed good to very good intraclass correlation coefficients (ICC = 0.87–0.99) in adults (25). In children, a mobility study in boys revealed an ICC in the range of 0.61 to 0.96 (26). The examiner applied the device paravertebrally along the spine or, in case of children with obesity, directly onto the processi spinosi. Therefore, the spine was marked from the seventh cervical vertebra (C7) to the first sacral vertebra (S1) to ensure high accuracy for the repetition of the measurement. The spinal curvature was measured in three positions of upright stand, maximal (spinal) flexion and maximal (spinal) extension. Three measurements were performed in each position. For further analyzes, the mean of the two measurements with the smallest variation was used. Between the lines through S1 and C7, representing the spinal inclination, the range in maximal flexion and maximal extension was defined as the total range of motion (ROM) of the spine (23, 27).

#### Physical fitness

Physical fitness was defined by a 20 m shuttle run. Children were asked to run back and forth from one line to the other (20 m) in the time between two acoustic signals. The frequency of the tone starts with an initial running speed of 8.5 km/h, increasing in stages by 0.5 km/h every minute (28). Stages were counted until the child could no longer maintain the speed. In their systematic review, Artero et al. (29) demonstrated that the 20 m shuttle run is a reliable assessment for cardiorespiratory fitness in children and adolescence and the shuttle run proved to have a good test-retest reliability when applied in 6–16 years-old children (*r* = 0.89) (28). It has been recommended for use in childrens` physical education classes because of its reliability and practicability (30).

#### Back pain

The Young Spine Questionnaire (YSQ) has previously been used to assess the occurrence of non-specific BP (lumbar, thoracic and cervical pain) in children. It was chosen based on its well-proven quality criteria and cohort fit and for reasons of practicability in this relatively large cohort (31). Since the questionnaire was only available in Danish, the questionnaire first had to be translated and checked for quality criteria (32). The children were interviewed about their back health by sports scientists. Answering the questions about the frequency of cervical, thoracic or lumbar pain with “often” or “once in a while” was defined as suffering from non-specific BP. The ICC of this item has been shown to be 0.88 (32).

### Statistical analysis

The population characteristics and standard values were described by calculating the mean and standard deviation. In addition, a paired T-test was performed to analyze the differences in the development from baseline to follow-up and between the sexes. To determine the relationship between physical fitness at baseline and spinal flexibility at follow-up, a linear regression analysis was performed. Furthermore, a logistic regression analysis was carried out to examine the association of spinal flexibility and physical fitness at baseline with non-specific BP at follow-up. The statistical models of causal relationships between the exposure and outcome variables were determined by Directed Acyclic Graphs (DAG's), using the free software “DAGitty” (33). Based on these results, adjustments were made for total effects concerning sex, age, Body Mass Index (BMI; classified in percentiles according to 34), spinal flexibility and physical fitness at baseline and follow-up. The statistical analysis was performed with Stata 15 and the statistical significance was set at ≤0.05.

## Results

### Population characteristics and standard values

A total of 1,264 children were invited to take part in the medical- and fitness screenings in 2014, of whom 540 children had a written consent to participate. 138 children were ill or relocated at the day of examination. Finally, 402 children had a complete baseline data set and thereof, 238 children continued to participate in 2018. A detailed description of the recruitment process and participation is shown in the flow chart (Figure 1). The population characteristics are shown in Table 1. Over four years, the children developed a higher overall ROM. In total, 39% of the children reported to suffer from BP “often” or “once in a while” at follow-up. In particular, an increased prevalence of neck pain occurred with 31.9% followed by thoracic pain (12,7%) and lumbar pain (8.4%).

**Table 1:** Population characteristics at baseline and follow-up.

Variables	N	2014		2018		Difference 2014–2018		
		Mean	SD	Mean	SD	Mean	SD	*P*-value
Sex (male, %)	238	48.3						
Age (y)	237	7.4	0.3	11.4	0.3	4.0	0.1	
Height (m)	226	1.3	0.5	1.5	0.7	0.23	0.3	
Weight (kg)	226	25.6	4.1	40.2	8.2	14.6	5.1	
BMI (kg/m^2^)	226	16.1	1.9	18.0	3.0	2.0	1.7	<0.001
Normal (%)		81		73				
Overweight (%)		8		12				
Obese (%)		7		2				
Z-BMI^a^	214	−0.1	0.9	−0.3	1.0	0.2	0.6	<0.001
CRF (stages)	224	4.6	1.7	6.5	2.1	1.9	1.7	<0.001
ROM overall (degree)	238	122.49	19.82	139.62	21.74	17.13	23.72	<0.001
Flexion (degree)	238	91.76	16.1	99.41	15.27	7.65	17.79	<0.001
Extension (degree)	238	−30.73	11.01	−40.20	10.8	−9.48	13.89	<0.001
Back pain (%)					39.1			
neck pain (%)					31.9			
thoracic pain (%)					12.7			
lumbar pain (%)					8.4			

^a^ Categorization of BMI is based on the KiGGS (German Health Interview and Examination Survey for Children and Adolescents) reference values (adjusted for age, sex and height). BMI indicates body mass index (according to 34); CRF, cardiorespiratory fitness (1 stage ≙ 1 min); ROM, range of motion of the overall spine; SD, standard deviation; Z-; standardized values based on the KiGGs reference values.

The development of the spinal flexibility from baseline to follow-up is presented in Table 2. In all areas, the overall flexibility at follow-up has significantly increased compared to baseline. This development was mainly due to the increase in girls' flexibility over the four years, as shown in the Supplementary Tables 2, 3. The spinal flexibility was significantly higher in girls than boys at age 10–12 years. In contrast, at baseline only the lumbar range of motion was characterized by significant gender differences.

**Table 2:** Spinal flexibility at baseline and follow-up.

Parameter	2014		2018		Difference 2014–2018		
	Mean	SD	Mean	SD	Mean	SD	*P*-value
Overall spine							
Upright (U)	−0.84	3.38	−1.28	3.36	−0.43	4.15	0.11
Flexion (F)	90.92	15.85	98.13	14.81	7.22	17.2	<0.001
Extension (E)	−31.57	10.97	−41.48	10.85	−9.1	13.68	<0.001
Range of motion (F-U)	91.76	16.1	99.41	15.27	7.65	17.8	<0.001
Range of motion (E-U)	−30.72	11.01	−40.2	10.8	−9.48	7.71	<0.001
Full range of motion (F-E)	122.49	19.82	139.62	23.72	17.13	23.72	<0.001
Thoracic spine							
Upright (U)	34.76	9.33	32.74	10.2	−2.03	10.94	0.005
Flexion (F)	56.67	7.02	55.65	7.81	−1.01	7.81	0.05
Extension (E)	38.41	14.35	32.58	13.01	−5.83	18.79	<0.001
Range of motion (F-U)	21.9	9.79	22.92	10.39	1.01	12.83	0.22
Range of motion (E-U)	3.64	14.63	−0.15	12.78	−3.8	18.7	0.002
Full range of motion (F-E)	18.26	14.94	23.07	13.66	4.81	19.36	<0.001
Lumbar spine							
Upright (U)	−31.52	10.61	−29.07	8.36	2.46	10.7	<0.001
Flexion (F)	32.01	10.36	36.2	7.69	4.19	10.41	<0.001
Extension (E)	−38.8	15.77	−43.1	10.13	−4.3	16.4	<0.001
Range of motion (F-U)	63.54	11.61	65.27	9.01	1.73	12.41	0.03
Range of motion (E-U)	−7.27	13.32	−14.03	10.76	−6.76	14.84	<0.001
Full range of motion (F-E)	70.81	16.81	79.3	12.03	8.49	17.81	<0.001
Pelvic tilt							
Upright (U)	19.42	9.43	19.98	7.01	−2.43	9.69	<0.001
Flexion (F)	52.36	15.4	55.06	15.56	2.7	16.23	0.01
Extension (E)	−6.01	19.3	−10.94	12.18	−4.92	20.63	<0.001
Range of motion (F-U)	32.94	15.76	38.08	16.56	5.13	17.27	<0.001
Range of motion (E-U)	−25.43	17.44	−27.92	11.62	−2.49	20.19	0.06
Full range of motion (F-E)	58.37	24.88	65.99	21.93	7.62	27.33	<0.001

### Baseline physical fitness and the development of spinal flexibility

One stage-increase in shuttle run performance at baseline was associated with a 3.75 degree higher spinal flexibility at follow-up (95%CI 2.19–5.3 degree, *p* < 0.001) as shown in Table 3. Furthermore, Table 3 depicts the predictive association of spinal flexibility and physical fitness with non-specific BP four years later. There was no significant correlation with BMI (B [95%CI]−0.71 [−2.25–0.83] per unit increase in BMI, *p* = 0.37).

**Table 3:** Association of spinal flexibility and cardiorespiratory fitness at baseline with back pain at follow-up.

		Spinal flexibility at baseline (per 1 degree)			Cardiorespiratory Fitness at baseline (per 1 stage)	
Dependent Variable	Model	OR (95% CI)	*P*-value	Model	OR (95% CI)	*P*-value
Back pain at follow-up	1	0.98 (0.97 to 0.99)	0.032	2	1.13 (0.96 to 1.34)	0.128

1 Adjusted for BMI and CRF at baseline and gender (defined by a DAG analysis).

2 Adjusted for age and gender (defined by a DAG analysis).

CI, Confidence Interval; CRF, cardiorespiratory fitness (1 stage ≙ 1 min); DAG, Directed Acyclic Graphs.

### Association of baseline spinal flexibility and physical fitness with development of non-specific back pain

**Figure 2:**
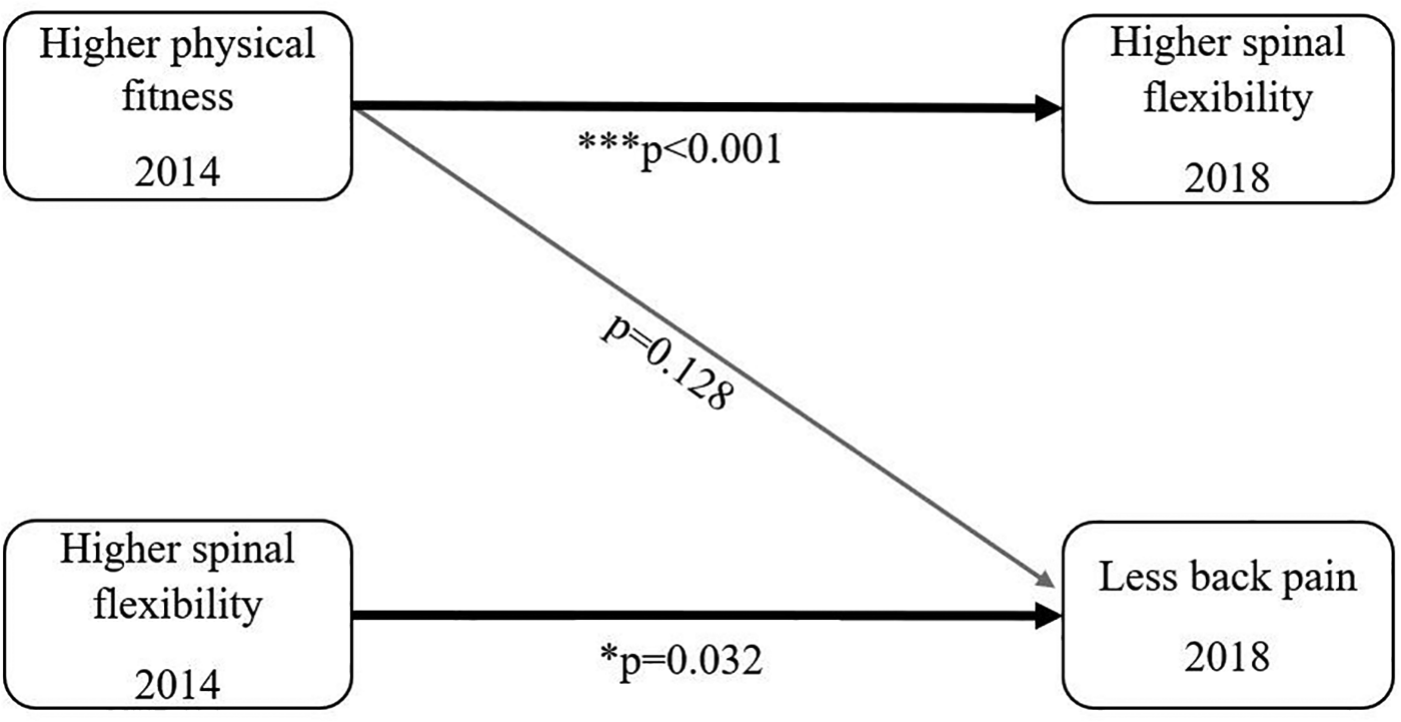
Overview of the results.

Children with an increase in spinal flexibility by one degree had a 2% lower odds for non-specific BP at follow-up (OR [95% CI] 0.98 [0.97–0.99] per 1 degree increase, *p* = 0.032). Little evidence for an association between physical fitness and the presence of non-specific BP at follow-up (2018) was found (OR [95% CI] 1.13 [0.96–1.34] per 1 stage increase, *p* = 0.128). The associations between physical fitness, spinal flexibility and BP for the time points are summarised in Figure 2. BMI had no significant influence on the development of BP (OR [95% CI] 1.04 [0.89–1.20] per 1 unit increase in BMI, *p* = 0.63).

## Discussion

The children have developed a higher spinal flexibility from baseline to follow-up, which corresponds to an age-related normal development (35). Almost every third child reported to suffer “once in a while” or “often” from BP at age of 10–12 years. Furthermore, our findings demonstrate that a better shuttle run performance at baseline is associated with an increased spinal flexibility at follow-up. In turn, a higher spinal flexibility was related to a lower susceptibility for BP four years later. Each degree increase in spinal flexibility at baseline resulted in a reduced odds of non-specific BP by 2%. No direct association between physical fitness performance at baseline and non-specific BP at follow-up was found.

Higher physical fitness performance in early childhood is associated with better spinal flexibility four years later. This finding builds on our cross-sectional findings from the baseline assessment (19), indicating that physical fitness at young age is predictive for the development of spinal flexibility during childhood. Nearly three decades ago, a case-control study in adolescent school children gave first evidence for a positive correlation between the weekly amount of physical activity and spinal flexibility, measured by forward bending (36). A more recent study confirmed that physical activity and, in particular, reduced sedentary behavior is beneficial for thoracic spine mobility in young adults (37). Most importantly, our results demonstrate that children with a better spinal flexibility at baseline were less likely to suffer from non-specific BP four years later. The risk to develop non-specific BP was reduced by 2% per one degree increased spinal flexibility at baseline. Our results are also in line with a smaller-sized study (38) revealing that a lower lumbar ROM at baseline contributes to future low BP in adolescent girls but not boys. In comparison, a larger cohort-study (39) found little evidence for an inverse association between spinal flexibility and low BP in adolescents. However, in their study the cross-sectional design and the controversially discussed sit-and-reach test to assess spinal flexibility have to be considered. According to Mayorga-Vega et al. (40) the sit-and-reach test is not an appropriate tool to measure lumbar extensibility (*r* = 0.16 to 0.35). In children it is likely that an acute overload of the active and passive structures of the spine induce pain more than degenerative processes (20). Limited flexibility is often accompanied by a weak musculature, as restricted flexibility allows for stability of the spine even with low muscular strength. On the other hand, a greater ROM can be explained by a stronger and therefore more coordinated and resilient musculature (trunk muscles as well as muscles attached to the pelvis) (41), possibly conditioned from a higher level of physical activity. No significant association between the shuttle run performance at baseline and non-specific BP at follow-up was found. The systematic review from Lardon et al. (42) concluded that the role of cardiorespiratory fitness in the development of BP in emerging adults is unclear. In a cross-sectional study, little evidence was found for an inverse association between shuttle run performance and BP in 10-years old children (41). In contrast, the aforementioned study from Andersen et al. (39) demonstrated that adolescents with a high maximal aerobic capacity were at less risk to develop BP compared to their unfit peers. However, the association did not remain significant after adjustment for the performance of muscle endurance (39). A potential explanation for our insignificant finding might be a short lifetime exposure for development of BP in prepubertal children, in consideration of the rising prevalence of BP in late adolescence and early adulthood (7). In addition, risk factors to develop non-specific BP are likely to be multifactorial and might manifest over time as children mature. Furthermore, cardiorespiratory fitness seems to act as a proxy measure for back muscle endurance with respect to the development of non-specific BP (42). Most importantly, our results demonstrate that increased physical fitness induces higher spinal flexibility, which, in turn, is related to reduced complaints of non-specific BP during childhood development.

### Limitations

Our findings need to be interpreted in light of some limitations. 164 children out of 402 were lost to follow-up (40.8%), which might have led to biased estimates. The population characteristics of the loss to follow-up group differed from the follow-up group in weight, BMI and performance in the shuttle run, but not in sex, height, spinal flexibility and BP (Supplementary Table 1). A standard tool to measure the spinal flexibility are x-rays (42). A high correlation between x-ray measurements as the gold standard and MediMouse has been shown (24), offering a cost-effective and harmless alternative for practical use. Furthermore, psychosocial health was not examined, although considered as an important risk factor for the development of BP in adolescence (43). In this study, the pain intensity and localization were not classified. Nevertheless, we achieved a good estimate for the prevalence of general BP in our cohort of children. A consistent risk factor for BP are previous episodes of back pain in emerging adulthood (10), indicating the importance of examination of even little pain to evaluate future back pain. The occurrence of non-specific BP was prospectively assessed at the age of 10–12 years at follow-up and, thus, adverse causality cannot be excluded. Further, by means of our examination we were not able to distinguish between persistent and transient non-specific BP. Screening for specific causes of BP, such as scoliosis and other spine deformations, was not performed due to time-related reasons in the school setting. However, the chosen methods to assess spinal flexibility and non-specific BP are considered as the most valid and feasible tools for a population-based screening approach in school settings (24–26). The prospective design and large sample size of prepubertal children make this a unique analysis of the interrelation between physical fitness, spinal flexibility and development of BP during childhood development.

## Conclusions

In conclusion, our findings demonstrate the predictive value of spinal flexibility for the development of non-specific BP in childhood. Children with limited spinal flexibility were at higher risk to develop BP later in life. A higher initial physical fitness was related to increased spinal flexibility after four years follow-up. It appears that physical activity and fitness have high potential as preventive strategies to directly improve spinal flexibility and, thereby, reduce the prevalence of non-specific BP and associated musculoskeletal disorders later in life. Analysis of spinal flexibility may proof to be a valid diagnostic screening tool in children to identify individuals at risk of developing BP and initiate physical activity programs to reduce the burden of BP in adolescence and adulthood as a long-term goal.

## What was already known about this topic

Back pain is a common health care burden and often originates in childhood
Childhood and adolescence back pain persists into adulthood

## What this study adds

Spinal flexibility is a predictor for back pain during childhood development
Promotion of physical fitness has the potential to improve spinal flexibility in children
Physical fitness can prevent development of back pain in children by improving spinal flexibility

## Data Availability

The raw data supporting the conclusions of this article will be made available by the authors, without undue reservation.

## References

[1] Hoy D Bain C Williams G March L Brooks P Blyth F A systematic review of the global prevalence of low back pain. Arthritis Rheum. (2012) 64(6):2028–37. 10.1002/art.34347 22231424

[2] Wu A March L Zheng X Huang J Wang X Zhao J Global low back pain prevalence and years lived with disability from 1990 to 2017: estimates from the global burden of disease study 2017. Ann Transl Med. (2020) 8(6):299. 10.21037/atm.2020.02.175 32355743 PMC7186678

[3] Schofield D Zeppel MJB Tanton R Veerman J Kelly ME Passey ME Informal caring for back pain: overlooked costs of back pain and projections to 2030. Pain. (2020) 161(5):1012–18. 10.1097/j.pain.0000000000001788 31895264

[4] Wieser S Horisberger B Schmidhauser S Eisenring C Brügger U Ruckstuhl A Cost of low back pain in Switzerland in 2005. Eur J Health Econ. (2011) 12(5):455–67. 10.1007/s10198-010-0258-y 20526649 PMC3160551

[5] Ellert U Neuhauser H Roth-Isigkeit A. Schmerzen bei kindern und jugendlichen in deutschland: prävalenz und inanspruchnahme medizinischer leistungen. Ergebnisse des kinder- und jugendgesundheitssurveys (KiGGS). Bundesgesundheitsblatt Gesundheitsforschung Gesundheitsschutz. (2007) 50(5-6):711–17. 10.1007/s00103-007-0232-8 17514455

[6] Jeffries LJ Milanese SF Grimmer-Somers KA. Epidemiology of adolescent spinal pain: a systematic overview of the research literature. Spine. (2007) 32(23):2630–37. 10.1097/BRS.0b013e318158d70b 17978666

[7] Du Y Knopf H Zhuang W Ellert U Pain perceived in a national community sample of German children and adolescents. Eur J Pain. (2011) 15(6):649–57. 10.1016/j.ejpain.2010.11.009 21177129

[8] Kamper SJ Yamato TP Williams CM. The prevalence, risk factors, prognosis and treatment for back pain in children and adolescents: an overview of systematic reviews. Best Pract Res Clin Rheumatol. (2016) 30(6):1021–36. 10.1016/j.berh.2017.04.003 29103547

[9] Hestbaek L Leboeuf-Yde C Kyvik KO. Is comorbidity in adolescence a predictor for adult low back pain? A prospective study of a young population. BMC Musculoskelet Disord. (2006) 7:29. 10.1186/1471-2474-7-29 16539740 PMC1431536

[10] Øiestad BE Hilde G Tveter AT Peat GG Thomas MJ Dunn KM Risk factors for episodes of back pain in emerging adults. A systematic review. Eur J Pain. (2020) 24(1):19–38. 10.1002/ejp.1474 31433541

[11] Franz C Wedderkopp N Jespersen E Rexen CT Leboeuf-Yde C. Back pain in children surveyed with weekly text messages—a 2.5 year prospective school cohort study. Chiropr Man Therap. (2014) 22(1):35. 10.1186/s12998-014-0035-6 25414789 PMC4237741

[12] Raspe H. Gesundheitsberichterstattung des bundes: Rückenschmerzen. Berlin: Robert Koch Institut (2012).

[13] Maher C Underwood M Buchbinder R. Non-specific low back pain. Lancet. (2017) 389(10070):736–47. 10.1016/S0140-6736(16)30970-9 27745712

[14] MacDonald J Stuart E Rodenberg R. Musculoskeletal low back pain in school-aged children: a review. JAMA Pediatr. (2017) 171(3):280–87. 10.1001/jamapediatrics.2016.3334 28135365

[15] Lallukka T Viikari-Juntura E Raitakari OT Kähönen M Lehtimäki T Viikari J Childhood and adult socio-economic position and social mobility as determinants of low back pain outcomes. Eur J Pain. (2014) 18(1):128–38. 10.1002/j.1532-2149.2013.00351.x 23813840

[16] Smith SM Sumar B Dixon KA. Musculoskeletal pain in overweight and obese children. Int J Obes (Lond). (2014) 38(1):11–5. 10.1038/ijo.2013.187 24077005 PMC3884137

[17] Paulis WD Silva S Koes BW van Middelkoop M. Overweight and obesity are associated with musculoskeletal complaints as early as childhood: a systematic review. Obes Rev. (2014) 15:52–67. 10.1111/obr.12067 23941399

[18] Wedderkopp N Kjaer P Hestbaek L Korsholm L Leboeuf-Yde C. High-level physical activity in childhood seems to protect against low back pain in early adolescence. Spine J. (2009) 9(2):134–41. 10.1016/j.spinee.2008.02.003 18495545

[19] Imhof K Faude O Strebel V Donath L Roth R Zahner L. Examining the association between physical fitness, spinal flexibility, spinal posture and reported back pain in 6 to 8 year old children. J Nov Physiother. (2015) (5):5. 10.4172/2165-7025.1000274

[20] Beynon AM Hebert JJ Lebouef-Yde C Walker BF. Potential risk factors and triggers for back pain in children and young adults. A scoping review, part I: incident and episodic back pain. Chiropr Man Therap. (2019) 27:58. 10.1186/s12998-019-0280-9 31827766 PMC6862727

[21] Robalo L Cruz E Nunes C. Epidemiology of non-specific back pain in children and adolescents: a systematic review of observational studies. J Nov Physiother. (2015) 05(03). 10.4172/2165-7025.1000266

[22] Joergensen AC Lucas R Hestbaek L Andersen PK Nybo Andersen A-M. Early-life programming of pain sensation? Spinal pain in pre-adolescents with pain experience in early life. Eur J Pediatr. (2019) 178(12):1903–11. 10.1007/s00431-019-03475-9 31624948

[23] Mannion AF Knecht K Balaban G Dvorak J Grob D. A new skin-surface device for measuring the curvature and global and segmental ranges of motion of the spine: reliability of measurements and comparison with data reviewed from the literature. Eur Spine J. (2004) 13:122–36. 10.1007/s00586-003-0618-8 14661104 PMC3476568

[24] Bistritschan E Delank S Winnekendonk G Eysel P. Oberflächenmessverfahren (medimouse) versus röntgenfunktionsaufnahmen zur beurteilung der lumbalen wirbelsäulenbeweglichkeit. Z Orthop Ihre Grenzgebiete. (2003) 141:141–X59. 10.1055/s-2003-821954

[25] Topalidou A Tzagarakis G Souvatzis X Kontakis G Katonis P. Evaluation of the reliability of a new non-invasive method for assessing the functionality and mobility of the spine. Acta Bioeng and Biomechanics. (2014) 16:117–24. 10.5277/abb140114 24707905

[26] Kellis E Adamou G Tzilios G Emmanouilidou M. Reliability of spinal range of motion in healthy boys using a skin-surface device. J Manipulative Physiol Ther. (2008) 31(8):570–76. 10.1016/j.jmpt.2008.09.001 18984239

[27] idiag. MediMouse®: Software Handbuch. Fehraltorf: idiag (2012).

[28] Léger LA Mercier D Gadoury C Lambert J. The multistage 20 metre shuttle run test for aerobic fitness. J Sports Sci. (1988) 6:93–101. 10.1080/02640418808729800 3184250

[29] Artero EG España-Romero V Castro-Piñero J Ortega FB Suni J Castillo-Garzon MJ Reliability of field-based fitness tests in youth. Int J Sports Med. (2011) 32:159–69. 10.1055/s-0030-1268488 21165805

[30] van Mechelen W Hlobil H Kemper HCG. Validation of two running tests as estimates of maximal aerobic power in children. Eur J Appl Physiol Occup Physiol. (1986) 55:503–06. 10.1007/BF00421645 3769907

[31] Lauridsen HH Hestbaek L. Development of the young spine questionnaire. BMC Musculoskelet Disord. (2013) 14:185. 10.1186/1471-2474-14-185 23758965 PMC3689606

[32] Nyirö L Potthoff T Siegenthaler MH Riner F Schweinhardt P Wirth B. Translation and validation of the German version of the young spine questionnaire. BMC Pediatr. (2021) 21(1):359. 10.1186/s12887-021-02804-y 34429090 PMC8383347

[33] Textor J van der Zander B Gilthorpe MS Liskiewicz M Ellison GT. Robust causal inference using directed acyclic graphs: the R package “dagitty”. Int J Epidemiol. (2016) 45(6):1887–94. 10.1093/ije/dyw341 28089956

[34] Cole TJ Bellizzi MC Flegal KM Dietz WH. Establishing a standard definition for child overweight and obesity worldwide: international survey. Br Med J. (2000) 320:1240–43. 10.1136/bmj.320.7244.1240 10797032 PMC27365

[35] Thuma M. Von der bedeutung gesundheitspräventiver maßnahmen für wiener volksschulkinder, vor allem statische und dynamische haltung bzw. Motorik betreffend; ausgehend vom modellprojekt “bewegtes lernen—das wiener modell” [Masterthesis]. Wien: (2007).

[36] Salminen JJ Oksanen A Mäki P Pentii J Kujala UM. Leisure time physical activity in the young: correlation with low-back pain, spinal mobility and correlation with low-back pain, spinal mobility and trunk muscle strength in 15-year-old school children. Int J Sports Med. (1993) 14:406–10. 10.1055/s-2007-1021200 8244608

[37] Heneghan NR Baker G Thomas K Falla D Rushton A. What is the effect of prolonged sitting and physical activity on thoracic spine mobility? An observational study of young adults in a UK university setting. BMJ Open. (2018) 8(5):e019371. 10.1136/bmjopen-2017-019371 29730619 PMC5942425

[38] Kujala UM Taimela S Oksanen A Salminen JJ. Lumbar mobility and low back pain during adolescence: a longitudinal three-year follow-up study in athletes and controls. Am J Sports Med. (1997) 25(3):363–68. 10.1177/036354659702500316 9167818

[39] Andersen LB Wedderkopp N Leboeuf-Yde C. Association between back pain and physical fitness in adolescents. Spine. (2006) 31(15):1740–44. 10.1097/01.brs.0000224186.68017.e0 16816772

[40] Mayorga-Vega D Merino-Marban R Viciana J. Criterion-Related validity of sit-and-reach tests for estimating hamstring and lumbar extensibility: a meta-analysis. J Sports Sci Med. (2014) 13(1):1–14. 24570599 PMC3918544

[41] Kendall FP. Muscles: testing and function, with posture and pain, 5th edn. Baltimore: Lippincott Williams & Wilkins (2005).

[42] Lardon A Leboeuf-Yde C Le Scanff C. Is back pain during childhood or adolescence associated with muscle strength, muscle endurance or aerobic capacity: three systematic literature reviews with one meta-analysis. Chiropr Man Therap. (2015) 23:21. 10.1186/s12998-015-0065-8 26185617 PMC4504178

[43] Potthoff T Bruin Ed Rosser S Humphreys BK Wirth B. A systematic review on quantifiable physical risk factors for non-specific adolescent low back pain. J Pediatr Rehabil Med. (2018) 11(2):79–94. 10.3233/PRM-170526 30010152

