# Supplementary material for: Prospective associations of physical fitness with spinal flexibility in childhood: implications for primary prevention of non-specific back pain
Source: Front Pediatr. 2023 Jul 4;11:1180690. doi: 10.3389/fped.2023.1180690 (PMC10352488; doi:10.3389/fped.2023.1180690)
Supplement: Supplementary file 1 [file Table1.pdf]

Supplementary Table 1: Dropouts at follow-up

Variables	Without Follow-Up			With Follow-up			Difference		
	N	Mean	SD	N	Mean	SD	Mean (95% CI)	SE	P-value
**Sex**	1355			238					0.246
**Age (y)**	1063	7.45	0.39	237	7.42	0.35	-0.25 (-0.79 to 0.28)	0.27	0.355
**Height (m)**	1059	126.48	5.86	237	126.09	4.98	-0.40 (-1.20 to 0.41)	0.41	0.333
**Weight (kg)**	1059	26.78	5.60	237	25.75	4.20	-1.03 (-1.79 to -0.28)	0.39	0.008
**BMI (kg/m^2)**	1059	16.62	2.56	237	16.13	1.93	-0.49 (-0.84 to -0.15)	0.18	0.005
**CRF (stages)**	1027	4.03	1.58	237	4.60	1.73	0.57 (0.34 to 0.79)	0.12	<0.001
**ROM overall (degree)**	164	124.45	12.43	238	124.45	18.43	-1.96 (-5.80 to 1.88)	1.96	0.316
**Back pain**	447			238					0.356

BMI indicates body mass index (according to Cole et al., 2000); CRF, cardiorespiratory fitness (1 stage ≙ 1 min); ROM, range of motion of the overall spine; SD, standard deviation.

T-tests were used to analyze the group differences, Mann-Whitney-U for differences between sex and back pain groups.
