# Supplementary material for: Prospective associations of physical fitness with spinal flexibility in childhood: implications for primary prevention of non-specific back pain
Source: Front Pediatr. 2023 Jul 4;11:1180690. doi: 10.3389/fped.2023.1180690 (PMC10352488; doi:10.3389/fped.2023.1180690)
Supplement: Supplementary file 2 [file Table2.pdf]

Supplementary Table 2: Sex specific spinal flexibility at baseline

	**Female**	**Male**	**P-value**	**Cohen's d**
**Parameter**	**Mean (95% CI)**	**Mean (95% CI)**		
**Overall spine**				
Upright (U)	-1.63 (-2.19 tp-1.07)	-0.004 (-0.64 to 0.63)	<0.001	0.49
Flexion (F)	90.52 (87.28 to 93.77)	91.34 (88.95 to 93.73)	0.69	0.05
Extension (E)	-33.04 (-35.1 to -30.98)	-30 (-31.87 to -28.13)	0.03	0.28
Range of motion (F-U)	92.15 (88.83 to 95.48)	91.34 (88.95 to 93.74)	0.7	0.05
Range of motion (E-U)	-31.41 (-33.48 to -29.33)	-29.99 (-31.90 to -28.09)	0.32	0.13
Full range of motion (F-E)	123.561 (119.60 to 127-52)	121.34 (118.21 to 124.47)	0.39	0.11
**Thoracic spine**				
Upright (U)	33.66 (31.34 to 35.48)	35.95 (34.44 to 37.46)	0.06	0.25
Flexion (F)	56.51 (55.29 to 57.73)	56.83 (55.49 to 58.18)	0.72	0.05
Extension (E)	37.52 (34.78 to 40.27)	39.36 (36.92 to 41.79)	0.33	0.13
Range of motion (F-U)	22.85 (20.91 to 24.79)	20.89 (19.33 to 22.44)	0.12	0.2
Range of motion (E-U)	3.87 (1.06 to 6.67)	3.41 (0.93 to 5.89)	0.81	0.03
Full range of motion (F-E)	18.98 (16.17 to 21.8)	17.48 (14.88 to 20.07)	0.44	0.1
**Lumbar spine**				
Upright (U)	-33.84 (-35.57 to -32.1)	-29.06 (-31.09 to -27.03)	<0.001	0.46
Flexion (F)	31.8 (29.73 to 33.88)	32-23 (30.59 to 33.87)	0.75	0.04
Extension (E)	-41.77 (-44.57 to -38.97)	-35.62 (-38.44 to -32.79)	0.003	0.4
Range of motion (F-U)	65.64 (63.53 to 67.76)	61.29 (59.27 to 63.31)	0.004	0.38
Range of motion (E-U)	-7.93 (-10.51 to -5.36)	-6.56 (-8.78 to -4.33)	0.43	0.1
Full range of motion (F-E)	73.58 (70.53 to 76.62)	67.85 (64.88 to 70.82)	0.008	0.35
**Pelvic tilt**				
Upright (U)	21.12 (19.6 to 22.64)	17.6 (15.74 to 19.45)	0.004	0.38
Flexion (F)	52.18 (49.4 to 54.96)	52.55 (49.73 to 55.37)	0.86	0.02
Extension (E)	-4.27 (-8.18 to -0.37)	-7.87 (-10.82 to -4.92)	0.15	0.19
Range of motion (F-U)	31.06 (28.21 to 33.92)	34.95 (32.12 to 37.78)	0.57	0.25
Range of motion (E-U)	-25.39 (-28.92 to -21.86)	-25.47 (-28.18 to -22.76)	0.97	0.005
Full range of motion (F-E)	56.46 (51.64 to 61.27)	60.42 (56.29 to 64.55)	0.22	0.16
