# Supplementary material for: Prospective associations of physical fitness with spinal flexibility in childhood: implications for primary prevention of non-specific back pain
Source: Front Pediatr. 2023 Jul 4;11:1180690. doi: 10.3389/fped.2023.1180690 (PMC10352488; doi:10.3389/fped.2023.1180690)
Supplement: Supplementary file 3 [file Table3.pdf]

Supplementary Table 3: Standard values of spinal flexibility at follow-up

	Female	Male	P-value	Cohen’s d
**Parameter**	**Mean (95% CI)**	**Mean (95% CI)**		
**Overall spine**				
Upright (U)	-1.3 (-1.87 to -0.72)	-1.26 (-1.91 to -0.61)	0.93	0.01
Flexion (F)	100.22 (97.72 to 102.72)	95.9 (93.06 to 98.74)	0.02	0.29
Extension (E)	-43.64 (-45.6 to -41.69)	-39.17 (-41.07 to -37.27)	0.001	0.42
Range of motion (F-U)	101.52 (98.98 to 104.06)	97.16 (94.19 to 100.12)	0.03	0.29
Range of motion (E-U)	-42.35 (-44.36 to -40.33)	-37.91 (-39.73 to -36.1)	0.001	0.42
Full range of motion (F-E)	143.87 (140.1 to 147.63)	135.07 (131.08 to 139.06)	0.002	0.41
**Thoracic spine**				
Upright (U)	32.76 (31.0 to 34.52)	32.71 (30.75 to 34.67)	0.97	0.005
Flexion (F)	55.21 (53.79 to 56.63)	56.13 (54.71 to 57.54)	0.37	0.12
Extension (E)	33.89 (31.55 to 36.23)	31.18 (28.87 to 33.55)	0.11	0.21
Range of motion (F-U)	22.45 (20.59 to 24.31)	23.41 (21.49 to 25.33)	0.48	0.09
Range of motion (E-U)	1.13 (-1.22 to 3.49)	-1.53 (-3.79 to 0.73)	0.11	0.21
Full range of motion (F-E)	21.32 (18.82 to 23.81)	24.94 (22.2 to 27.36)	0.04	0.27
**Lumbar spine**				
Upright (U)	-31.81 (-33.32 to -30.30)	-26.14 (-27.47 to -24.81)	<0.001	0.72
Flexion (F)	34.82 (33.36 to 36.27)	37.68 (36.41 to 38.95)	0.004	0.38
Extension (E)	-45.77 (-47.52 to -44.01)	-40.25 (-42.04 to -38.46)	<0.001	0.57
Range of motion (F-U)	66.63 (64.84 to 68.42)	63.82 (62.42 to 65.22)	0.02	0.32
Range of motion (E-U)	-13.96 (-15.97 to -11.94)	-14.11 (-16 to -12.23)	0.91	0.02
Full range of motion (F-E)	80.59 (78.5 to 82.67)	77.93 (75.66 to 80.2)	0.09	0.22
**Pelvic tilt**				
Upright (U)	19.25 (18.03 to 20.46)	14.56 (13.37 to 15.74)	<0.001	0.71
Flexion (F)	58.22 (55.46 to 60.98)	51.67 (48.90 to 54.44)	0.001	0.43
Extension (E)	-11.18 (-13.4 to -8.96)	-10.68 (-12.89 to -8.47	0.75	0.41
Range of motion (F-U)	38.97 (35.98 to 41.97)	37.11 (34.1 to 40.14)	0.39	0.11
Range of motion (E-U)	-30.43 (-32.55 to -24.31)	-25.23 (-27.22 to -23.25)	<0.001	0.46
Full range of motion (F-E)	69.4 (65.46 to 73.34)	62.35 (58.42 to 66.28)	0.01	0.33
